# Evaluating the efficacy and immunological impact of combined ICIs and SBRT in HCC: A narrative literature review

**DOI:** 10.1016/j.ctro.2026.101106

**Published:** 2026-01-11

**Authors:** Lara Caglayan, Oliver Blanck, Judit Boda-Heggemann, Thomas Brunner, Cas Stefaan Dejonckheere, Dan G. Duda, Elke Firat, Maria Hawkins, Julian Philipp Layer, Christina Leitzen, Alejandra Mendez Romero, Gabriele Niedermann, Younèss Nour, Falk Röder, Gustavo Renato Sarria, Davide Scafa, Marta Scorsetti, Shari Wiegreffe, Anca-L. Grosu, Eleni Gkika

**Affiliations:** a Department of Radiation Oncology, University Hospital Bonn, Venusberg-Campus 1, 53127 Bonn, Germany; b Department of Radiation Oncology, University Medical Center Schleswig-Holstein, Campus Kiel, Arnold-Heller-Straße 3, Haus 50, 24105 Kiel, Germany; c Department of Radiation Oncology, University hospital Mannheim, Theodor-Kutzer-Ufer 1-3, 68167 Mannheim, Germany; d Department of Radiation Oncology, Medical University of Graz, Auenbruggerplatz 14, 8036 Graz, Austria; e E. L. Steele Laboratories for Tumor Biology, Department of Radiation Oncology, Massachusetts General Hospital and Harvard Medical School, 55 Fruit Street Boston, MA 02114, USA; f Department of Radiation Oncology, Medical Center-University of Freiburg, Faculty of Medicine, 79106 Freiburg, Germany; g Gray Institute for Radiation Oncology and Biology, University of Oxford, Oxford, Old Road Campus Research Building Off Roosevelt Drive, Oxford OX3 7DQ, UK; h Institute of Experimental Oncology, University Hospital Bonn, Venusberg-Campus 1, 53127 Bonn, Germany; i Department of Radiation Oncology, Erasmus MC - Daniel den Hoed Cancer Center, Dr. Molewaterplein 40, 3015 GD Rotterdam, The Netherlands; j Department of Radiation Oncology, Faculty of Medicine, Medical Center-University of Freiburg, Robert-Koch-Straße 3, 79106 Freiburg, Germany; k Department of Radiotherapy and Radiation Oncology, Paracelsus Medical University, Landeskrankenhaus, Müllner Hautpstrasse 48, 5020 Salzburg, Austria; l Radiotherapy and Radiosurgery Department, IRCCS Humanitas Research Hospital, Rozzano, 20089 Milan, Italy; m Department of Biomedical Sciences, Humanitas University, Pieve Emanuele, 20072 Milan, Italy

**Keywords:** Radiation, HCC, SBRT, Immunotherapy

## Abstract

Hepatocellular carcinoma (HCC) represents one of the leading contributors to cancer-related deaths, with the majority of patients diagnosed at stages where curative treatment is no longer possible. Combining stereotactic body radiotherapy (SBRT) with immune checkpoint inhibition (ICI) has gained increasing attention as a therapeutic approach. Beyond its ability to provide high local tumor control (LC), SBRT can provoke immunogenic tumor cell death, promote antigen release and presentation, and modulate the tumor microenvironment in ways that enhance systemic antitumor immunity.

In this narrative review, we outline the scientific rationale for integrating SBRT with ICIs, discuss mechanistic and translational findings and summarize results from key clinical trials. The currently available data indicate a synergistic interaction, most notably reflected in improved survival and response rates. Nevertheless, variability in dose and fractionation schedules, treatment sequencing, and patient characteristics complicates interpretation. Well-designed prospective studies are needed to establish optimal protocols and identify predictive biomarkers to guide patient selection.

## 1 Introduction

Hepatocellular carcinoma (HCC) is the sixth most common and lethal malignancy worldwide [1]. In 2020 it caused an estimated 906,000 new cases and 830,000 fatalities [2]. Risk factors commonly associated with HCC encompass infection by the hepatitis B virus and hepatitis C virus. Consumption of alcohol, and non-alcoholic steatohepatitis associated with metabolic syndrome or diabetes mellitus is becoming a more frequent risk factor in the West [2], [3]. Persistent inflammation is a well-known factor that contributes to the promotion and aggravation of malignancies, as more than 90% of HCC cases develop in the context of chronic liver injury and inflammation [4]. Its treatment remains a significant challenge due to often advanced stage at diagnosis, as over 70% of HCC are diagnosed as unresectable [5]. Treatment is commonly guided by the Barcelona Clinic Liver Cancer (BCLC) clinical stage [6].

Current treatment options for unresectable HCC are multimodality and include transarterial chemoembolization (TACE), transarterial radioemboliztation (TARE; or selective internal radiotherapy [SIRT]), EBRT (external beam radiotherapy) including stereotactic body radiotherapy (SBRT) [7] microwave-/radiofrequency ablation (MWA/RFA) or systemic therapies [6]. TACE is considered one of the preferred locoregional treatments for unresectable HCC [8]. However, its use may be limited in patients with significantly impaired liver function, e.g., Child-Pugh-Score (CP-Score) C, due to the increased risk of liver decompensation. In patients with portal vein HCC thrombosis (PVTT), TACE is not categorically contraindicated but requires careful patient selection.

SBRT provides a focused and potent radiation therapy approach capable of targeting and potentially controlling HCC growth and size [9], [10]. Therefore, SBRT is currently reserved mainly for localized HCC not amenable to other local treatment options [11], [12], [13] and for the palliative setup [7]. Recently, the integration of immunotherapy, particularly ICIs, has opened new perspectives in HCC management. Immune checkpoint pathways, particularly CTLA-4 (Cytotoxic T-Lymphocyte Antigen 4) and PD-1/PD-L1 (Programmed Cell Death Protein 1/Programmed Cell Death Ligand 1), play a significant role in modulating the anti-HCC immune response against HCC tumor cells [14], [15], [16]. Several trials have already investigated the combination of SBRT and ICI in the setting of advanced and unresectable HCC.

This narrative review aims to summarize evidence on the use of combined SBRT and ICI, it will examine the rationale for immune-targeted therapy of HCC, discuss key studies on combination therapy and evaluating the current state of research and clinical outcomes as well as future prospects.

## 2 Immunotherapy

**Fig. 1:**
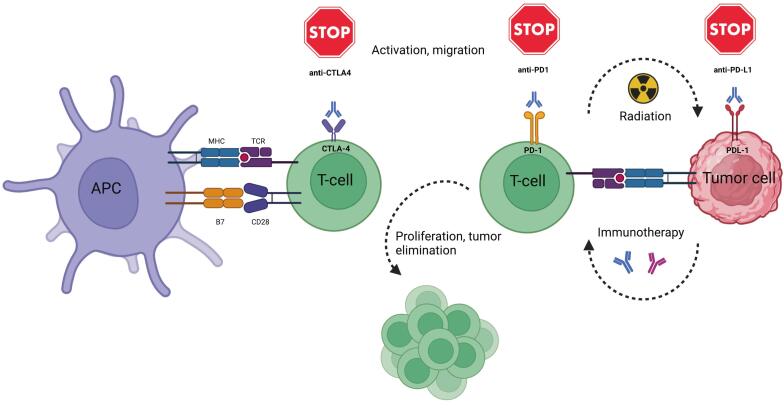
Blockade of CTLA-4 and PD-1 pathways. Blocking CTLA-4 leads to the activation and expansion of more T-cell clones while reducing the immunosuppressive effects of regulatory T-cells. Inhibiting the PD-1 pathway reinvigorates exhausted antitumor T-cells. CTLA-4 refers to cytotoxic T-lymphocyte-associated antigen 4; MHC to major histocompatibility complex; PD-1 to programmed cell death protein 1; and PD-L1 to programmed death-ligand 1; TCR refers to the T-cell receptor; B7 molecules are costimulatory proteins found on antigen-presenting cells. They interact with the CD28 receptor on T-cells to promote activation, or with the CTLA-4 receptor to inhibit the immune response (Created in BioRender.com).

The most thoroughly documented immune checkpoint molecules include PD-1/PD-L1, CTLA-4, LAG3 (Lymphocyte-activation gene 3), and TIM3 (T-cell immunoglobulin and mucin-domain containing-3), of which PD-1/PD-L1 and CTLA-4 are validated targets for HCC therapy [17], [18]. These mAbs against these molecules' function by antagonizing the regulatory pathways that suppress T cell mediated immune responses [19], [20], [21], [22] by blocking the interaction between checkpoint proteins on cancer cells and those on immune cells. As a result, immune cells can recognize cancer cells, leading to an activated immune response [12] (Fig. 1).

Key clinical ICIs-trials are CheckMate 040 [23], KEYNOTE 224 [24], Checkmate 459 [25] and IMBrave150 [26]. The CheckMate 040 trial was a phase 1/2 multicenter study investigating the efficacy and safety of nivolumab, a monoclonal PD-1 antibody, in advanced HCC. Participants had CP scores of up to 7 in the dose-escalation phase (where the dose was gradually increased to determine the maximum tolerated dose) and up to 6 in the dose-expansion phase (where the fixed dose was given to a larger group to confirm its efficacy and safety). The overall response rate (ORR) in the dose-expansion phase was 20%, in the dose-escalation phase was 15%. The trial led to the FDA (Food and Drug Administration) approval of nivolumab in 2017 [27]. Importantly, nivolumab was approved for use not only in patients with CP A liver function but also for those with class B, while the majority of ICI are available for CP A only. An extension of the CheckMate 040 trial investigated the combined use of nivolumab and ipilimumab. The trial demonstrated a manageable safety profile, stable responses, and showed potential for improved clinical outcomes for patients with advanced HCC [28]. The subsequent CheckMate 459 trial compared nivolumab directly with sorafenib in patients with CP A liver function and found that nivolumab did not demonstrate a significant advantage over sorafenib. A higher ORR of 15% was noted with nivolumab compared to 7%, yet this did not result in a significant improvement in overall survival (OS), which was 16.4 months for nivolumab versus 14.7 months (p = 0.075). As a result, the use of nivolumab was suggested mainly for CP B patients as an alternative therapy [28]. The KeyNote 224 trial examined the safety and effectiveness of pembrolizumab in patients who had previously been treated with sorafenib. The study reported an ORR of 18.3%, with 3.8% of participants experiencing a complete response and 14.4% showing a partial response. The median response duration was 21.0 months, and the disease control rate was 61.5%. Additionally, progression free survival (PFS) was 4.9 months, and OS was 13.2 months [29].

The IMbrave150 trial compared the combination therapy against the standard sorafenib treatment in patients with unresectable HCC who had not received previous systemic treatment. The results showed significantly better OS and PFS outcomes with the combination therapy compared to the standard sorafenib treatment, boosting the 1-year OS from 54.6% to 67.2%. The ORR for those treated with the combination therapy was 27.3% (p < 0.001).

The HIMALAYA trial [30] evaluated the combination of tremelimumab and durvalumab as a first-line treatment for unresectable HCC. This phase 3 trial involved 1171 patients who were randomly assigned to one of three treatment groups: the STRIDE regimen (a single priming dose of tremelimumab plus durvalumab every 4 weeks), durvalumab alone, or sorafenib. The results demonstrated that the STRIDE regimen achieved a median OS of 16.43 months, compared to 13.77 months for sorafenib, with a statistically significant HR ratio of 0.78(p < 0.01). Additionally, durvalumab alone achieved a median OS of 16.56 months, demonstrating noninferiority to sorafenib. Finally, 19.6% of patients treated with STRIDE remained alive at 5 years, compared to 9.4% among those treated with sorafenib

## 3 SBRT

SBRT is a non-invasive, highly precise method of radiation therapy that aims to administer high doses of radiation in a limited number of fractions. During the last decade, SBRT has shown remarkable efficacy in HCC patients achieving high local control (LC) rates of 90–95% at 2 years [[31], [32], [33]]. While SBRT had not been formally integrated into the previous versions of the BCLC treatment algorithm, the 2025 update of the BCLC guidelines now includes SBRT as a treatment option for unresectable or locally HCC when other local therapies are unsuitable [29]**.** The ESMO (European Society for Medical Oncology) guideline [34] also recommend SBRT as a bridging option prior to transplantation, as an ablative treatment in BCLC A when resection or thermal ablation are not feasible, in BCLC B alone or in combination with TACE, and in BCLC C with macrovascular invasion either alone or combined with systemic therapy. In February 2025, the European Association for the Study of the Liver (EASL) introduced SBRT as a guideline-recommended treatment for HCC (EASL Clinical Practice Guidelines on the management of HCC) [35].

The NCCN guidelines [12], [36]endorse SBRT particularly for unresectable HCC or in cases where surgery is not an option. In addition, the 2022 ASTRO (American Society of Radiation Oncology) guideline highlights SBRT as a definitive treatment, a bridging strategy to transplantation, and for the management of residual disease after TACE.

Patients with moderate liver function are deemed appropriate for SBRT since they have a lower likelihood of hepatic decompensation [37]. The ability to treat these lesions with SBRT in a fragile patient group is largely dependent on how much of the liver tissue can be spared from radiation to maintain adequate liver function [38], [39], [40]. The AAPM Liver Working Group (American Association of physicists in Medicine) investigated the potential relationship between HCC tumor control and various dose-fractionation schemes for primarily HCC. Their findings did not establish a definite dose–response relationship, even for high radiation doses (BED ≥ 100 Gy) [41]. While higher doses have shown an association with improved LC in univariate analysis, this correlation often loses its significance in multivariate analyses [41], [42], [43]. In the last prospective multicenter trial led by Kimura et. al [44], a cohort of 36 patients with previously untreated, solitary primary HCC was enrolled to evaluate the efficacy and safety profile of SBRT. The protocol specified a radiation dose of 40 Gy in 5 fractions. At the three-year follow-up mark, LC rates were documented at 90%, with OS at 78%. Notably, adverse events classified as CTCAE 4.0 (Common Terminology Criteria for Adverse Events**)** Grade 3 or higher were observed in 11% of the cohort, including duodenal ulceration, dyspnea coupled with hypoxia, ascites, hepatic failure, and thrombosis of the portal vein. Additionally, a deterioration of two or more points in the CP score was recorded in one-third of patients.

In the latest systematic review of Bae et. al [45] seventeen observational studies were analyzed between 2003 and 2019, which included 1889 patients with HCC treated with up to 9 fractions of SBRT. This analysis showed that the 3-year and 5-year OS after SBRT were 57% and 40%, respectively. Additionally, the LC at 3 years and 5 years were 84% and 82%. HCC size was a critical determinant for LC and was significantly associated with OS outcomes. An individual patient data analysis highlighted 5-year LC and OS rates of 79% and 25%, respectively, with improved OS linked to factors such as a HCC size smaller than 3 cm, location in the Eastern region, and a CP score of B7 or lower.

Xi et. Al [46] conducted a randomized study comparing SBRT and RFA in 166 patients. In which SBRT achieved significantly higher local progression-free survival (LPFS) compared to RFA (p = 0.014), with 2-year LPFS rates of 92.7% and 75.8%, respectively. PFS (37.6 vs. 27.6 months; p = 0.190) and OS remained comparable between both groups (2-year OS 97.6% vs. 93.9%; p = 0.830). Fu et. al [47] conducted a multicenter retrospective study comparing resection, ablation, and SBRT for solitary HCC ≤ 5 cm across 985 patients. The 3-year recurrence-free survival rates were 66.8% after resection, 49.8% after ablation, and 56.4% after SBRT (p < 0.001). The 3-year OS rates were comparable across all three modalities at 89.0%, 89.2%, and 88.8% (p = 0.590). Subgroup analysis showed that SBRT achieved similar recurrence-free survival to resection in tumors < 3 cm (p = 0.205) and in patients with poorer liver function (p = 0.330). Notably, SBRT demonstrated a significant advantage over ablation for tumors located adjacent to intrahepatic vessels (p = 0.031).

## 4 Possible immunmodulatory effects of SBRT

The rationale behind combining SBRT with immunotherapy in HCC treatment lies in the synergistic immunomodulatory potential of these two treatment modalities [48]. Radiotherapy exerts multiple anti-tumor effects at the local level through immune-mediated mechanisms, engaging both the innate and adaptive immune systems in the process [49]. SBRT can initiate immunogenic cell death in tumor cells by effectively targeting and killing radiosensitive CD8-T-cell lymphocytes. Simultaneously, it spares the more radiation-resistant regulatory T-cells [50]. Radiotherapy-induced immunogenic cell death triggers the release of tumor- associated antigens (TAAs) and of damage-associated molecular pattern (DAMPs) [51].These TAAs are subsequently captured and displayed by antigen-presenting cells (APCs) within the lymph nodes [52]. As APCs process and present these antigens, they activate CD4 + T helper (TH) cells, which further mature APCs through the interaction of CD40 ligand. The mature APCs then promote the differentiation and proliferation of CD8 + CTLs (Cytotoxic T lymphocytes) by producing type I interferons and interleukins facilitating a TH1 response [53]. Mature dendritic cells can present tumor antigens to CD4 + T-cells through the MHC class II pathway, additionally they can cross-present antigens to CD8 + T-cells via the MHC (Major Histocompatibility Complex) I pathway. This dual mechanism activates CTLs, allowing them to infiltrate tumors and target cancer cells for destruction. Concurrently the radiation-induced immunogenic modulation increases the expression of MHC I molecules and TAAs on tumor cells, enhancing the visibility of these cells to the immune system [51]. The endogenous antitumor immune response is influenced by several factors, including the immunogenicity of cancer antigens; radiotherapy can enhance the expression of these “antigens” and promote their translocation to the cell surface [54]. Tumors also secrete immunosuppressive cytokines, such as TGF-β and IL-10, and certain radiation doses can further amplify their production [55]. Additionally, T-cell function is inhibited through signaling by negative costimulatory molecules such as PD-1 and CTLA-4, which are upregulated in response to radiotherapy-induced T-cell activation [14], [56].

## 5 Combination ICI and SBRT in clinical trials and dose regimes

We reviewed several published studies on the concomitant use of SBRT with ICIs and highlighted both published and ongoing clinical trials.

A phase 2 clinical trial by Li et al. [57] included 21 patients with a CP A/B liver function. From April 2020 to August 2022, these patients received 200 mg of camrelizumab on the initial day of SBRT treatment, followed by subsequent administrations every three weeks. SBRT was applied daily, with dosage ranging from 30-50 Gy in 5 fractions (median dose 40 Gy). OS was 85.7% at six months, 76.2% at nine months and 59.9% at twelve months. Grade 3 treatment-related toxicities were recorded in five patients (23.8%).

In the first prospective randomized phase I trial Juloori et al. [58] combined SBRT (40 Gy in 5 fractions) with ICIs (nivolumab alone or with ipilimumab) in patients with advanced or unresectable HCC. Seven patients were treated with combination therapy, while six patients underwent treatment with nivolumab. The median follow-up time was 42.7 months. In the group receiving combination therapy, four patients experienced Grade 3 hepatotoxicity, and three patients in the nivolumab group had similar side effects. The combination therapy group showed improved outcomes compared to nivolumab alone, with higher ORR (57% vs. 0%), longer PFS (11.6 vs. 2.7 months), and extended OS (41.6 vs. 4.7 months).

A phase 2 trial “START-FIT” by Chiang et. al [59] included patients with locally advanced HCC. Treatment involved TACE followed by SBRT with doses of 27.5–40 Gy in 5 fractions and then biweekly administration of ICI (Avelumab). Out of 33 enrolled patients over 50% were considered for curative treatment after the regimen. Grade 3 events or higher toxicities occurred in 33% of patients. 42% of patients had a radiological complete response according to mRECIST (modified Response Evaluation Criteria in Solid Tumors). Chen et al’s. [60] prospective, single-arm trial used a combination therapy of toripalimab and anlotinib (6 cycles) in patients with unresectable HCC after undergoing SBRT with doses of 3x8 Gy. The study enrolled 20 who were classified as CP A or B. The study showed a median PFS of 7.4 months, an ORR of 15%, and a disease control rate (DCR) of 50%. The OS was 61.1% at 18 months and 50.9% at 24 months with PFS of 39.3% and 19.7% for the same time periods, respectively.

A retrospective study from Ning etal. [61] included 76 patients who were diagnosed with advanced HCC and underwent treatment with ICI and antiangiogenic therapy. Of these, 33 patients were part of the radiation therapy (RT) group, while 43 were included in the non-RT group. The median PFS extended to 8.3 months in the RT group, contrasting with 4.2 months in the non-RT cohort (p < 0.001). The median OS was not reached in the RT group, compared to 9.7 months in the non-RT group (p < 0.002). Furthermore, the RT group demonstrated a substantially higher ORR (75.9%) and DCR (100%) relative to the non-RT group. The median follow-up duration was 15.5 months. For radiation therapy, 24 patients (82.8%) received IMRT (Intensity-Modulated Radiation Therapy)**,** with prescribed doses ranging from 30 to 60 Gy over 8–30 fractions, and 5 patients (17.2%) received SBRT afterwards, with prescribed doses ranging from 40 to 60 Gy over 8–10 fractions.

In a retrospective multicenter observational study by Ning et al, [62] 36 patients with advanced HCC who developed new lesions or experienced progression of existing lesions during treatment with ICIs received subsequent radiotherapy, either as SBRT or IMRT. SBRT was delivered with doses ranging from 24 to 50  Gy in 3–10 fractions, while IMRT was administered with doses between 30 and 70  Gy in 5–30 fractions. IMRT was used in 61.1% of patients and SBRT in 38.9%. Importantly, the study did not evaluate SBRT and IMRT as separate modalities. The median follow-up was 15.3 months. The PFS was 7.4 months and the median OS was 18.8 months. The ORR was 38.9%, and the DCR was 72.2%. Additionally, the median in-field PFS was 17.8 months and the out-field PFS was 7.9 months. The estimated 1- and 2-year LC rates were 67.1% and 31.9%, respectively. The median follow-up period was 15.3 months.

In the latest prospective study by Chiang et al. [63] patients first received TACE on day 1, followed by five fractions of SBRT on day 28, with a median dose of 35.0 Gy. Among the 63 patients included in the study, 49 (77.8%) underwent TACE prior to SBRT, while 14 (22.2%) were treated with SBRT and IO alone. The immunotherapy consisted of anti–PD-1 or anti–PD-L1 antibodies, including avelumab (52.4%), nivolumab (42.9%), and pembrolizumab (4.8%). The 3-year OS rate among patients who achieved complete response (CR) was 75.5%, significantly higher than the 28.1% observed in those who did not achieve CR (p < 0.001). CR patients also had a 3-year LC rate of 90.5% and a time-to-progression rate of 58.7%. CR was more likely in patients without vascular invasion and in those with tumors measuring ≤ 8 cm. A phase 3 randomized trial by Dawson et al. reported a statistically significant OS improvement for SRS followed by sorafenib compared with sorafenib alone (median OS 15.8 vs. 12.3 months; HR 0.72; p = 0.04) [64]

Beyond clinical outcomes, experimental work has shown that fractionated SBRT regimens interact more favorably with ICIs than single high-dose irradiation, enhancing systemic immune activation and abscopal effects [65], [66]. Translational studies in HCC further support this by demonstrating increased CD8 + T-cell infiltration and a shift toward a less immunosuppressive tumor microenvironment after SBRT [67].

## 6 Discussion and future directions

Recent studies have explored the combination of SBRT and ICIs in patients with unresectable HCC, most often using SBRT regimens in the range of 3 to 10 fractions, though no consensus on the optimal regimen has been reached. While these studies consistently reported prolonged OS and favorable response rates, a clear dose–response relationship for SBRT in this context has not been established yet (Table 1). [65] Preclinical data indicate that very high single doses can induce Trex1, degrading cytosolic DNA and suppressing immune activation [68]. Demaria et al. [69] proposed a model categorizing tumors as immunologically “hot” (T-cell inflamed) or “cold” (low antigenicity, poor infiltration), with implications for tailoring fractionation and immunotherapy choice.

**Table 1:** Summary of ongoing trials of immunotherapy combined with SBRT for unresectable and advanced HCC; HCC (Hepatocellular carcinoma), PVI (Portal vein invasion)

Trial number	Phase	HCC stage	Dose (Gy)/ Fractionation (Fx)	Immunotherapy	Enrollment
NCT04913480	II	unresectable HCC	27.5–50 Gy/5 Fx	Durvarlumab every two weeks, an additional dose one week before SBRT	37
NCT03817736	II	unresectable HCC	Not specified	TACE + SBRT followed by Durvalumab + Tremelimumab	33
NCT03316872	II	locally advanced HCC/unresectable HCC/metastatic HCC	Not specified; SBRT starting day 2 of the first cycle of pembrolizumab	Pembrolizumab every 3 weeks	30
NCT03482102	II	HCC/biliary tract tumor; advanced HCC/unresectable HCC/metastatic HCC	Not specified; SBRT during cycle 2 of Durvalumab + Tremelimumab	Durvalumab + Tremelimumab every 4 weeks	70
NCT05096715	I	unresectable HCC	Not specified; SBRT during cycle 1	Atezolizumab + Bevacizumab every 3 weeks (6 cycles)	20
NCT04785287	I/II	Multiple tumor malignancies including HCC	Not specified; SBRT on days 8–11 of cycle 2	Nivolumab administered every 4 weeks (4 cycles)	13
NCT06040177	I/II	unresectable HCC with PVI	25–40 Gy/5 Fx	Cardonilizumab (within 2 weeks after completion of radiation therapy; once every 3 weeks) + Lenvatinib (once a day until disease progression/toxicity)	30
NCT05286320	I/II	HCC with PVI	5 Fxs within 17 days	Pembrolizumab every 3 weeks, Lenvatinib every day for 2 years	27
NCT04988945	II	unresectable HCC	Not specified; TACE following SBRT	Durvalumab administered every 4 weeks, with a single high dose of Tremelimumab (300 mg) given initially	33
NCT05488522	I	Locally advanced HCC	17 Gy/3 Fxs	Atezolizumab + Bevazizumab every 3 weeks	18
NCT05396937	II	Metastatic HCC	25–50 Gy/5–10 Fxs	Atezolizumab and Bevacizumab. After completing radiotherapy, systemic therapy begins 48 h post-radiotherapy	42
NCT06339424	II	unresectable HCC	39.6–72.6 Gy in 22 fractions for tumors ≤ 1 cm from the hepatic hilum, bowel, and heart 30–66 Gy in 10 fractions for tumors > 1 cm from the hepatic hilum, bowel, and heart27.5–50 Gy in 5 fractions	Atezolizumab + Bevacizumab administered every 3 weeks	45

In the randomized trial by Juloori et al. [58], which was terminated early due to slow accrual, the addition of SBRT to nivolumab and ipilimumab showed a trend to improved ORR, PFS and OS compared to Nivolumab alone. Despite these promising findings, the current evidence is limited. Most available studies are small single-arm, or retrospective, with substantial heterogeneity in inclusion criteria such as CP scores, tumor burden, and prior therapies and in response criteria. While Chiang et al. [59] used both RECIST 1.1 and mRECIST, Chen et al [60] relied on iRECIST (immune Response Evaluation Criteria in Solid Tumors**)**. This heterogeneity complicates the assessment of LC and emphasizes incomparability across studies. In addition the timing of SBRT relative to ICI administration also varied considerably. Most trials administered SBRT sequentially, with ICIs initiated days to weeks after completion of radiotherapy. In contrast, Li et al. [57] applied ICI on the first day of SBRT..These different sequencing strategies may also influence immunologic priming and treatment efficacy, but have not yet been compared directly. The combination of SBRT and ICIs has demonstrated favorable systemic outcomes, including improved OS and ORR in several studies. In contrast, a consistent benefit regarding LC beyond that achieved with SBRT alone has not yet been established, as LC was either not reported or not assessed comparatively.

In summary, current evidence suggests that the combination of SBRT and ICIs may offer a synergistic effect in patients with unresectable HCC, with the most consistent improvements observed in OS. Based on available data, this combination appears particularly effective in patients with advanced HCC and MVI [64]. The use of doses below the ablative threshold appear sufficient to induce immunomodulatory effects that enhance the efficacy of ICIs. However, due to the limited number of prospective, randomized trials and the methodological heterogeneity of existing studies, firm conclusions cannot yet be drawn. Future trials are urgently needed to define optimal dose-fractionation schedules, treatment sequencing, and patient selection strategies for this promising combination approach.

## Data Availability Statement for this Work

Research data are stored in an institutional repository and will be shared upon request to the corresponding author.

## Funding

None.

## Declaration of Competing Interest

The authors declare that they have no known competing financial interests or personal relationships that could have appeared to influence the work reported in this paper.
